# Use of Bioceramic Sealers in Clinical Practice Among Dentists in Saudi Arabia: A Cross-Sectional Analysis

**DOI:** 10.7759/cureus.105484

**Published:** 2026-03-19

**Authors:** Amer M Almutairi, Abdullah A Aldafas, Turke Alabdalgder, Bader S Almutairi, Eithar I Alrosaa, Ghadeer Almutairi

**Affiliations:** 1 Department of Dentistry, Cyreen Clinics, Elaj Sudair Polyclinic Medical Company, Zulfi, SAU; 2 Department of Dentistry, Medical Center, King Fahad Security College, Riyadh, SAU; 3 College of Dentistry, Majmaah University, Majmaah, SAU

**Keywords:** bioceramic sealers, clinical adoption, dentist awareness, endodontic practice, kingdom of saudi arabia (ksa)

## Abstract

Background

Bioceramic sealers have recently gained attention in endodontic therapy due to their superior biological and physicochemical properties compared to traditional sealers. However, adoption of these sealers and awareness among the dental practitioners in Saudi Arabia remain underexplored. This study aims to assess the prevalence, knowledge, and patterns of bioceramic sealer use among dentists in Saudi Arabia and to identify factors influencing their clinical application.

Methods

It is a cross-sectional survey that was conducted among 75 dentists who practice in Saudi Arabia. All the data were collected using a structured questionnaire, which covered demographic characteristics, familiarity, knowledge, use patterns, advantages, and barriers related to bioceramic sealers. Associations between demographic variables and sealer awareness and use were analyzed using chi-square and logistic regression tests via IBM SPSS Statistics software, version 29.0.0 (IBM Corp., Armonk, NY, USA).

Results

Out of 75 dentists, most of them were familiar with the bioceramic sealers (65, 86.7%), and 49 (65.3%) reported that they were using them in practice. The main perceived advantages included biocompatibility (32.9%), antimicrobial effect (18.7%), and ease of use (17.4%). High cost (34.2%) and unfamiliarity (18.4%) were the most reported barriers. Use of bioceramic sealers was significantly associated with gender (p=0.030) and years of clinical experience (p=0.027). Although awareness was high across all groups, logistic regression showed no significant predictors of high knowledge (p > 0.05).

Conclusion

The dentists in Saudi Arabia demonstrated very high awareness but moderate use of the bioceramic sealers. Cost and limited training are the main obstacles to the broader adoption of these sealers. Expanding continuing education and improving material accessibility may enhance clinical integration and optimize endodontic outcomes using bioceramic sealers.

## Introduction

The treatment of root canals relies on the effective adaptation of the obturation materials within the canal system. The sealers of the root canal play an important role in the achievement of a hermetic seal, fill accessory canals, and serve as lubricants to help in the placement and entombment of bacteria [1]. There are various categories of these sealers, which include zinc oxide eugenol, calcium hydroxide, resin, and glass ionomer. Bioceramic sealers have emerged as a notable advancement in endodontic therapy [2]. These bioceramic sealers have further different types based on material, which include calcium silicate, phosphate, and other bioactive ceramic materials that offer a host of advantageous properties [3]. These properties include excellent biocompatibility, an alkaline pH with antimicrobial effects, dimensional stability without shrinkage, and their ability to bond chemically with dentin via hydroxyapatite formation, which reduces microleakage and fosters healing of tissues [4, 5]. Their hydrophilic nature allows them to set even in the presence of moisture, which is a frequent challenge in clinical practice [2]. Notably, many of these formulations are delivered via convenient premixed systems, which further simplify their use [5]. There are several in vitro and clinical studies that have underscored the benefits of bioceramic sealers. One of the recent clinical investigations showed that these sealers showed significantly lower microbial leakage and superior apical seal integrity as compared to the resin-based alternatives, alongside the reduced rates of postoperative pain [6]. Additionally, bioceramic sealers such as BioRoot RCS and Sure-Seal Root demonstrated strong antimicrobial activity against persistent endodontic pathogens, including *Porphyromonas gingivalis* and *Enterococcus faecalis*, attributable to hydroxyl ion release and their high pH environment [7]. Despite these advancements, adoption of the bioceramic sealers varies across different regions due to several factors such as practitioner familiarity, exposure to training, cost, and availability.

In Saudi Arabia, as the endodontic practices evolve continuously in line with global trends, there is a need to understand how widely these sealers are integrated into routine practice, dentists' awareness of their properties, and the clinical outcomes associated with their use. Therefore, there is a need for a cross-sectional analysis in order to focus on dentists in Saudi Arabia, which can shed light on current usage patterns, knowledge levels, and the determinants that will guide the choice of bioceramic sealers. This insight would inform targeted educational initiatives and guide future research tailored to local clinical realities. This study aims to assess the prevalence and patterns of bioceramic sealer use among dentists in Saudi Arabia. Also, as secondary objectives, it aims to: 1) Evaluate dentists’ perceived knowledge and awareness of the properties, advantages, and limitations of bioceramic sealers. 2) Identify the main factors associated with dentists’ choice of root canal sealers, with a focus on bioceramic types; 3) Assess the level of training and continuing education related to bioceramic sealer application among practitioners. 4) Explore perceived clinical outcomes, benefits, and challenges associated with the use of bioceramic sealers.

## Materials and methods

Study setting

A cross-sectional study was conducted between August 2025 and December 2025 in the Riyadh region, Saudi Arabia, with the aim of assessing the knowledge and awareness, prevalence, and utilization of bioceramic sealers among dentists in Saudi Arabia. The research involved both Saudi and non-Saudi dentists who were actively seeing patients during the study period. A total of 75 responses were collected through an online questionnaire distributed using a convenience sampling approach via social media and professional e-mail. Despite the fact that the calculated required sample size was 381. 

The sample size was calculated using the standard formula for cross-sectional studies, which is as follows:

 $n = \frac{Z^{2} \times p(1-p)}{d^{2}}$​

where Z represents the standard normal deviation corresponding to a 95% confidence level (1.96), p represents the estimated prevalence (0.5), and d represents the margin of error (0.05). The sample size was 381 participants.

A structured questionnaire was produced and reevaluated specifically for this study out of thorough literature on bioceramic sealers. The survey instrument was developed into two parts: the first part, which collected the demographic data, including age, gender, nationality, number of years of experience, specialty, and type of practice, while the second part assessed the participant's familiarity, perceived knowledge, clinical use, perceived advantages, and barriers pertaining to bioceramic sealers (Appendix A). The questionnaire was evaluated by two experts for content relevance, clarity, and suitability for the target population, therefore endorsing the face and content validity of the instrument. Prior to participation, informed consent was obtained electronically from all respondents, and participation was voluntary and anonymous. Potential confounding variables, including years of clinical experience, gender, specialty, and type of practice, were considered during the statistical analysis. These variables were included where appropriate to account for their possible influence on sealer use.

Inclusion criteria

Dentists (Saudi or non-Saudi) currently practicing in Saudi Arabia were eligible to participate. Dentists not engaged in active clinical practice during the study period were excluded.

Statistical analysis

The dataset was screened for missing values prior to analysis, and complete responses were included in the final analysis. Data were analyzed using IBM SPSS Statistics software, version 29.0.0 (IBM Corp., Armonk, NY, USA). Descriptive statistics were used to summarize categorical variables as frequencies and percentages. Associations between demographic variables (age group, gender, nationality, years of clinical experience, practice type, and specialty) and familiarity with bioceramic sealers, as well as current use of bioceramic sealers were assessed using the chi-square test. Fisher’s exact test was applied when expected cell counts were less than five. Binary logistic regression analysis was conducted to explore factors associated with higher knowledge of bioceramic sealers. A p-value < 0.05 was considered statistically significant.

## Results

Our study included 75 dentists for the assessment of prevalence and patterns of bioceramic sealer use in endodontic practice. Most respondents were aged between 25 and 34 years (39, 52.0%), followed by those under 25 years (14, 18.7%) and 35 to 44 years (14, 18.7%), while eight (10.7%) were above 45 years. The gender distribution was nearly balanced, with 39 (52.0%) females and 36 (48.0%) males. The majority were Saudi nationals (55, 73.3%), and 20 (26.7%) were non-Saudi. Regarding clinical experience, 29 (38.7%) had less than five years, 24 (32.0%) had five to 10 years, and 16 (21.3%) had 11 to 20 years, while six (8.0%) had over 20 years. Most dentists practiced in private settings (46, 61.3%), followed by university/academic (20, 26.7%) and government (nine, 12.0%) sectors. In terms of specialty, 44 (58.7%) were general dentists, 19 (25.3%) endodontists, and 12 (16.0%) belonged to other specialties (Table 1).

**Table 1:** Demographic characteristics of the participants (n=75)

		Frequency N (%)
Age	<25 Years	14 (18.7%)
	25–34 Years	39 (52.0%)
	35–44 Years	14 (18.7%)
	>45 Years	8 (10.7%)
Gender	Female	39 (52.0%)
	Male	36 (48.0%)
Nationality	Saudi	55 (73.3%)
	Non-Saudi	20 (26.7%)
Years of Clinical Experience	<5 Years	29 (38.7%)
	5–10 Years	24 (32.0%)
	11–20 Years	16 (21.3%)
	>20 Years	6 (8.0%)
Practice Type	Private	46 (61.3%)
	University/Academic	20 (26.7%)
	Government	9 (12.0%)
Specialty	General Dentist	44 (58.7%)
	Endodontist	19 (25.3%)
	Others	12 (16.0%)

**Figure 1:**
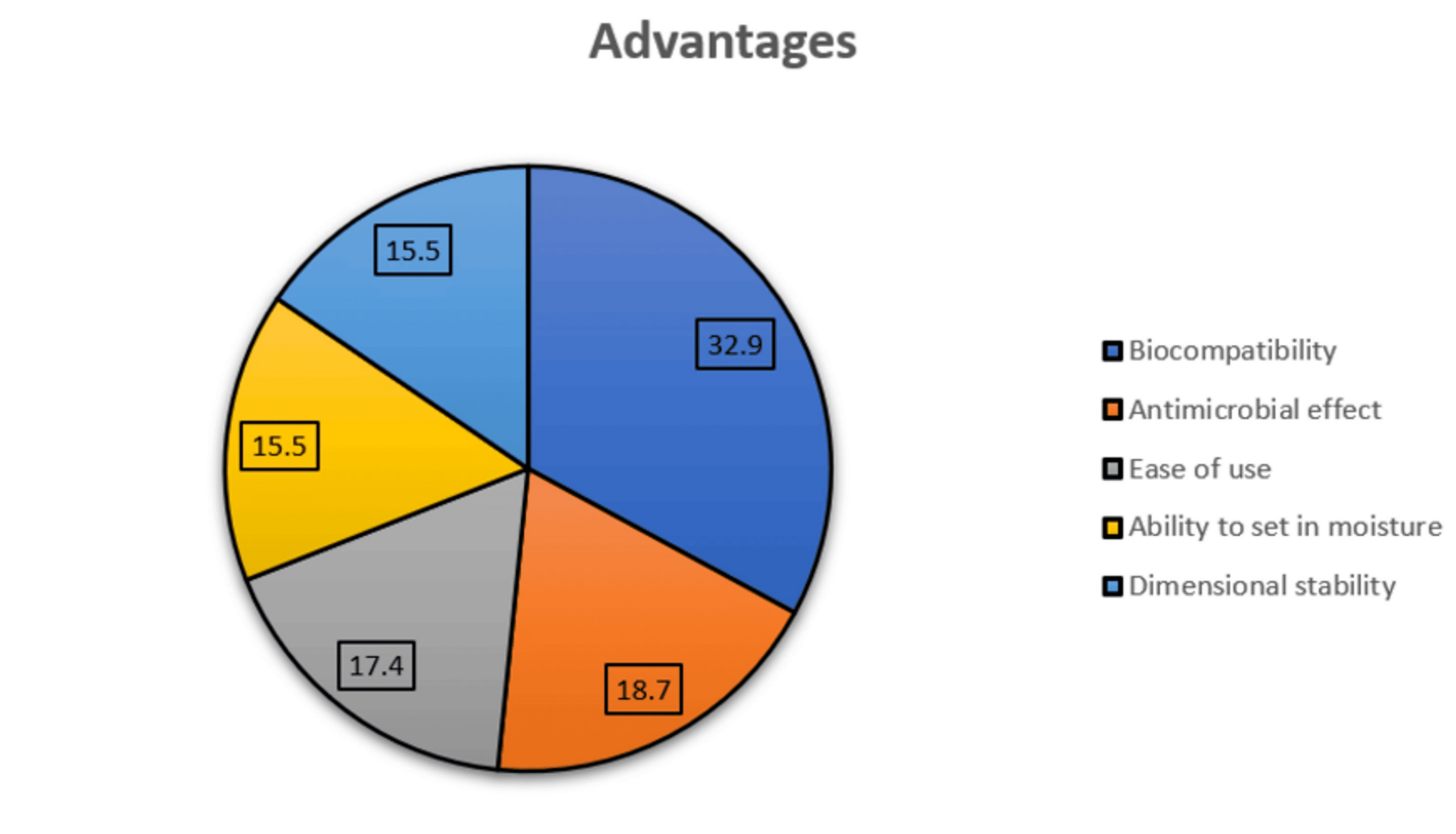
Reported advantages of bioceramic sealers among the participants Note: The data are reported in percentages (%).

Figure 1 shows the advantages of bioceramic sealers. The most frequently cited benefit was biocompatibility (32.9%), followed by antimicrobial effect (18.7%), ease of use (17.4%), ability to set in moisture (15.5%), and dimensional stability (15.5%).

Table 2 shows participants’ familiarity, knowledge, and practice patterns regarding bioceramic sealers. Most dentists were familiar with bioceramic sealers (65, 86.7%), while 10 (13.3%) were not. In terms of knowledge, 32 (42.7%) rated theirs as good and 21 (28.0%) as excellent, whereas 15 (20.0%) had fair and seven (9.3%) had poor knowledge. Over half of respondents currently use bioceramic sealers (49, 65.3%). Regarding frequency of use, 24 (32.0%) reported using them often, and 22 (29.3%) always. The single cone technique (38, 50.7%) was the most common obturation method. Most participants believed bioceramic sealers provide superior outcomes (47, 62.6%). More than half attended continuing education or training (40, 53.3%), while 35 (46.7%) had not.

**Table 2:** Familiarity, knowledge, and practice patterns of bioceramic sealers (n=75)

		Frequency N (%)
Familiar with Bioceramic Sealers	Yes	65 (86.7%)
	No	10 (13.3%)
Knowledge of Bioceramic Sealers	Poor	7 (9.3%)
	Fair	15 (20.0%)
	Good	32 (42.7%)
	Excellent	21 (28.0%)
Currently Use Bioceramic Sealers	Yes	49 (65.3%)
	No	26 (34.7%)
Frequency of Use Compared to Other Sealers	Rarely	13 (17.3%)
	Sometimes	16 (21.3%)
	Often	24 (32.0%)
	Always	22 (29.3%)
Most Common Obturation Technique with Bioceramic Sealers	Single Cones	38 (50.7%)
	Lateral Condensation	14 (18.7%)
	Warm Vertical Compaction	10 (13.3%)
	Others	13 (17.3%)
Opinion: Bioceramic Sealers Provide Superior Outcomes	Strongly Disagree	6 (8.0%)
	Disagree	7 (9.3%)
	Neutral	15 (20.0%)
	Agree	22 (29.3%)
	Strongly Agree	25 (33.3%)
Attended Continuing Education/Training on Bioceramic Sealers	Yes	40 (53.3%)
	No	35 (46.7%)

**Figure 2:**
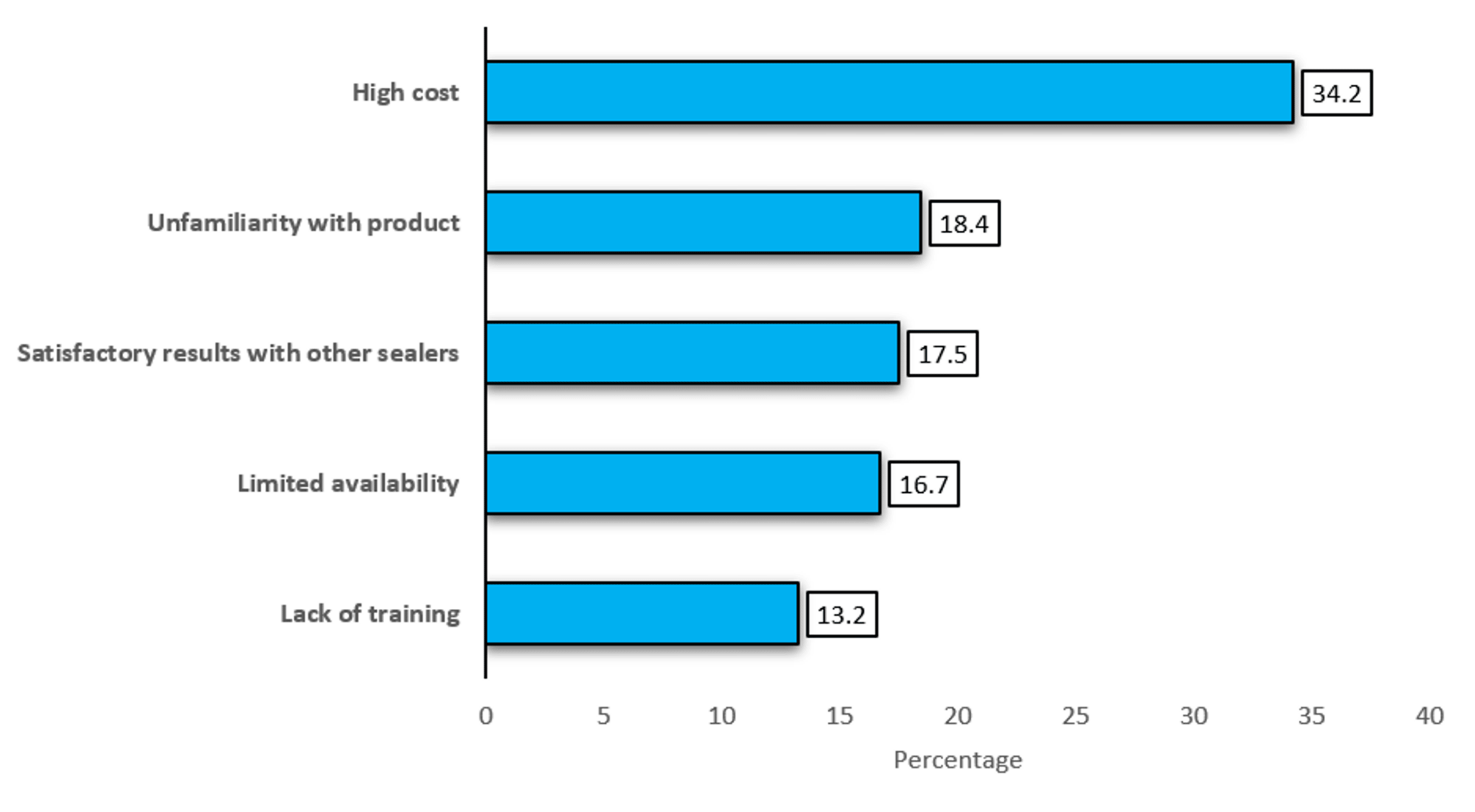
Reported barriers to the use of bioceramic sealers (n=75) Note: The data are reported in percentages (%).

Figure 2 shows the main challenges perceived by participants regarding the use of bioceramic sealers. The high cost (34.2%) was the most frequently reported barrier, followed by unfamiliarity with the product (18.4%), satisfactory results with other sealers (17.5%), limited availability (16.7%), and lack of training (13.2%).

Table 3 shows the association between demographic characteristics and awareness of bioceramic sealers among participants. Awareness was generally high across all subgroups, with no statistically significant associations observed. Among age groups, familiarity increased slightly with older age, from 78.6% in those <25 years to 100% among dentists >45 years (p = 0.452). Awareness was similar between females (84.6%) and males (88.9%) (p = 0.586), and between Saudi (85.5%) and non-Saudi (90.0%) dentists (p = 0.609). Familiarity was high across experience levels, ranging from 82.8% (<5 years) to 100% (>20 years) (p = 0.526). By practice type, awareness was highest in private (89.1%) and academic (90.0%) sectors compared to government (66.7%) (p = 0.170). Specialty also showed no significant difference (p = 0.429).

**Table 3:** Association between demographic variables and awareness with bioceramic sealers (n=75) Associations were assessed using the chi-square test. Fisher’s exact test was applied when expected cell counts were <5. χ²: chi-square statistic; df: degrees of freedom

		Aware of Bioceramic Sealers		Test Value (χ², df)	df	p-value
		Not Familiar n (%)	Familiar n (%)			
Age	<25 Years	3 (21.4%)	11 (78.6%)	χ² = 2.63	3	0.452
	25–34 Years	6 (15.4%)	33 (84.6%)			
	35–44 Years	1 (7.1%)	13 (92.9%)			
	>45 Years	0 (0.0%)	8 (100.0%)			
Gender	Female	6 (15.4%)	33 (84.6%)	χ² = 0.30	1	0.586
	Male	4 (11.1%)	32 (88.9%)			
Nationality	Non-Saudi	2 (10.0%)	18 (90.0%)	χ² = 0.26	1	0.609
	Saudi	8 (14.5%)	47 (85.5%)			
Years of Clinical Experience	<5 Years	5 (17.2%)	24 (82.8%)	χ² = 2.23	3	0.526
	5–10 Years	4 (16.7%)	20 (83.3%)			
	11–20 Years	1 (6.3%)	15 (93.8%)			
	>20 Years	0 (0.0%)	6 (100.0%)			
Practice Type	Private	5 (10.9%)	41 (89.1%)	χ² = 3.55	2	0.170
	University/Academic	2 (10.0%)	18 (90.0%)			
	Government	3 (33.3%)	6 (66.7%)			
Specialty	General Dentist	5 (11.4%)	39 (88.6%)	χ² = 1.69	2	0.429
	Endodontist	2 (10.5%)	17 (89.5%)			
	Others	3 (25.0%)	9 (75.0%)			

Table 4 shows the association between demographic characteristics and the current use of bioceramic sealers among participating dentists. Overall, 49 (65.3%) reported using bioceramic sealers, while 26 (34.7%) did not. Use increased with age, from 35.7% among those <25 years to 87.5% among those >45 years, though this difference approached but did not reach statistical significance (p = 0.054). A significant association was found with gender (p = 0.030), as males (77.8%) were more likely to use bioceramic sealers than females (53.8%). Similarly, years of clinical experience were significantly related to usage (p = 0.027); dentists with more than five years’ experience showed greater adoption. Nationality, practice type, and specialty were not significantly associated with use (all p > 0.05).

**Table 4:** Association Between Demographic Variables and Use of Bioceramic Sealers (n=75) Associations were assessed using the chi-square test. Fisher’s exact test was applied when expected cell counts were <5. χ²: chi-square statistic; df: degrees of freedom

		Use of Bioceramic Sealers		Test Value (χ²)	df	Sig. Value
		Not Using N (%)	Using N (%)			
Age	<25 Years	9 (64.3%)	5 (35.7%)	χ² = 7.65	3	0.054
	25–34 Years	12 (30.8%)	27 (69.2%)			
	35–44 Years	4 (28.6%)	10 (71.4%)			
	>45 Years	1 (12.5%)	7 (87.5%)			
Gender	Female	18 (46.2%)	21 (53.8%)	χ² = 4.73	1	0.030*
	Male	8 (22.2%)	28 (77.8%)			
Nationality	Non-Saudi	6 (30.0%)	14 (70.0%)	χ² = 0.26	1	0.609
	Saudi	20 (36.4%)	35 (63.6%)			
Years of Clinical Experience	<5 Years	16 (55.2%)	13 (44.8%)	χ² = 9.21	3	0.027*
	5–10 Years	5 (20.8%)	19 (79.2%)			
	11–20 Years	3 (18.8%)	13 (81.3%)			
	>20 Years	2 (33.3%)	4 (66.7%)			
Practice Type	Private	15 (32.6%)	31 (67.4%)	χ² = 0.34	2	0.842
	University/Academic	8 (40.0%)	12 (60.0%)			
	Government	3 (33.3%)	6 (66.7%)			
Specialty	General Dentist	18 (40.9%)	26 (59.1%)	χ² = 4.01	2	0.135
	Endodontist	3 (15.8%)	16 (84.2%)			
	Others	5 (41.7%)	7 (58.3%)			

Table 5 shows the binary logistic regression analysis identifying factors associated with high knowledge about bioceramic sealers among participants. None of the examined predictors demonstrated a statistically significant association (all p > 0.05). However, the current use of bioceramic sealers showed a positive trend (B = 1.017, p = 0.098), indicating that users were approximately 2.8 times more likely to report high knowledge compared to non-users (Exp(B) = 2.765, 95% CI: 0.830-9.209). Other demographic variables, including age (p = 0.703), gender (p = 0.256), nationality (p = 0.843), years of experience (p = 0.982), practice type (p = 0.974), and specialty (p = 0.46), were not significant predictors.

**Table 5:** Binary logistic regression analysis of factors associated with high knowledge about bioceramic sealers B: regression coefficient; SE: standard error; Exp(B): odds ratio; CI: confidence interval

	B	SE	Sig. (p-value)	Exp(B) (95% CI)
Age	0.171	0.448	0.703	1.187 (0.493 – 2.857)
Gender	-0.683	0.601	0.256	0.505 (0.156 – 1.640)
Nationality	0.151	0.76	0.843	1.162 (0.262 – 5.156)
Years of Clinical Experience	-0.01	0.445	0.982	0.990 (0.414 – 2.368)
Practice Type	0.015	0.458	0.974	1.015 (0.414 – 2.491)
Specialty (Other)	Ref	Ref	0.46	Ref
Specialty (General Dentist)	-0.986	0.874	0.259	0.373 (0.067 – 2.069)
Specialty (Endodontist)	-0.392	1.086	0.718	0.676 (0.080 – 5.675)
Current Use of Bioceramic Sealers	1.017	0.614	0.098	2.765 (0.830 – 9.209)

## Discussion

Root canal sealers are essential for the achievement of a tight seal and prevention of bacterial leakage [8]. Bioceramic sealers, which are composed mainly of calcium silicate and phosphate, offer superior biocompatibility, antimicrobial effects, dimensional stability, and chemical bonding with dentin [9]. They can be set in moisture and are easy to use. Previous studies have shown their strong antimicrobial activity and better sealing ability as compared to the resin-based sealers. However, adoption of these sealers varies due to the cost, familiarity, and the training [10]. This study aims to describe awareness, use, and factors associated with the reported utilization of bioceramic sealers among dentists in Saudi Arabia.

Notably, although awareness of bioceramic sealers was high (86.7%) in this study, actual clinical use was comparatively lower (65.3%), which suggested a gap between knowledge and practice. This discrepancy may reflect practical barriers within the Saudi healthcare context, which include higher material costs, limited availability in certain clinical settings, and procurement policies that favor conventional sealers, particularly in public institutions. Additionally, while dentists may be theoretically familiar with bioceramic sealers, inconsistent hands-on training and variable exposure during undergraduate and postgraduate programs may limit routine adoption. Similar disparities between awareness and utilization have been reported in previous studies, such as by Rajguru et al. (2025), which indicated that structural and system-level factors, rather than lack of knowledge alone, play a critical role in shaping clinical practice [11]. Another study by Garde et al. (2025) found that only 11.1% of participants utilized the bioceramic sealers despite high awareness [12]. Moreover, similar trends have been reported in other studies where dentists showed an increasing interest in bioceramic sealers due to their improved properties and clinical benefits. Another study showed that bioceramic sealers achieved better results since 90% of patients experienced periapical healing compared to 75% in the conventional group [13]. The growing awareness could be related to the continuous advancements in endodontic materials, the availability of training programs, and greater exposure through conferences and dental education.

The most commonly reported advantages of the bioceramic sealers were biocompatibility (32.9%), antimicrobial effect (18.7%), and ease of use (17.4%). These findings are consistent with the previous research, which showed that bioceramic sealers are biologically safe, promote tissue healing, and have good handling characteristics. Al-Haddad et al. (2016) show that bioceramic-based sealers were found to be biocompatible and comparable to other commercial sealers [1]. Other studies have also shown that they bond chemically to dentin, provide excellent sealing ability, and can set in moist conditions, making them suitable for routine clinical use [14]. These benefits explain why many dentists consider them superior to the conventional sealers, such as zinc oxide eugenol or resin-based types.

Despite these advantages, there are some barriers that continue to limit their wider use. The most frequently mentioned obstacle in this study was high cost (34.2%), which is followed by unfamiliarity with the product (18.4%) and satisfactory results with other sealers (17.5%). Limited availability (16.7%) and lack of training (13.2%) were also reported. These barriers are in line with previous studies by Kumara et al. (2025) and Rajguru et al. (2025), which have identified cost and accessibility as the main reasons for the slow adoption of bioceramic materials [15, 11]. The results suggest that while dentists recognize the benefits of bioceramic sealers, practical and financial factors still influence material selection.

In terms of the demographic factors, several significant associations were found for gender and years of clinical experience. Notably, male dentists and those with more than five years of experience were more likely to use the bioceramic sealers. This may be due to the higher clinical confidence, greater exposure to new materials, or increased financial independence among the experienced dentists, as shown in a previous study by Merfea et al. (2025) [16]. Moreover, the awareness level was high across all groups, showing that knowledge of bioceramic sealers has reached both new and experienced practitioners. These findings aligned with other studies, which found that experience plays a key role in the adoption of newer dental materials [17].

Although the awareness was very high, the binary logistic regression analysis showed that none of the demographic factors significantly predicted the high knowledge levels. However, dentists who are currently using the bioceramic sealers were almost three times more likely to report good knowledge compared to non-users. This suggests that hands-on clinical experience contributes to a better understanding of the material’s properties and advantages.

Regarding the obturation techniques, most of the participants who used bioceramic sealers preferred the single cone technique (50.7%), followed by lateral condensation (18.7%). This finding is consistent with the manufacturer's recommendations and previous studies (Zhang et al., 2025), as the single cone technique takes advantage of the sealer’s hydraulic expansion and simplifies the obturation process [18].

In addition, there are 53.3% of the participants had attended the training or continued the education session related to the bioceramic sealers, while 46.7% had not. Although a lack of training was not the main barrier, these results show that there is a need for more workshops and educational programs. Continuous education can improve both the confidence and clinical skills in handling bioceramic materials, especially among less experienced dentists.

Implications

There is a need for greater clinical integration of the bioceramic sealers in endodontic practice. Despite the high awareness, there are several barriers, such as the cost and limited training, which restrict the widespread use. Enhancing access to affordable materials and promoting education can improve adoption. The increased utilization of the bioceramic sealers may enhance treatment outcomes by providing better sealing ability, biocompatibility, and antimicrobial properties, ultimately improving long-term success rates in root canal therapy.

Limitations

There are several limitations of this study. The cross-sectional design of this study limited the ability to establish the causal relationships between the variables. The data were self-reported, which may introduce recall or response bias. The use of a convenience sampling approach and the relatively small sample size may also introduce selection bias and limit the generalizability of the findings. Additionally, the clinical outcomes and the long-term performance of the bioceramic sealers were not assessed. Also, a key limitation of this study is that knowledge was assessed using a self-reported scale, which reflects perceived rather than objective knowledge. 

Future recommendations

Future studies with larger and more diverse samples of dentists across different regions of Saudi Arabia are recommended to provide a more representative understanding of bioceramic sealer usage patterns. Additionally, multicenter studies and longitudinal research could help evaluate changes in awareness and clinical adoption over time. Further clinical studies assessing the long-term outcomes and effectiveness of bioceramic sealers compared with conventional sealers would also be valuable to guide clinical decision-making in endodontic practice.

## Conclusions

This study showed that there is high awareness and moderate reported use of bioceramic sealers among dentists in Saudi Arabia. Most of the participants have recognized their biological and handling advantages, and these are particularly biocompatibility and antimicrobial effects. However, the cost, limited availability, and the lack of training were the main barriers to wider use. There is a need for education programs, improving product accessibility, and reducing costs, which may support greater adoption of bioceramic sealers in endodontic practice. Further research is needed to better understand their impact on clinical outcomes and patient care in the region.

## Appendices

Appendix A

Bioceramic Sealers Questionnaire

Section 1: Demographic Information

Age □ <25  □ 25-34  □ 35-44  □ >45

Gender □ Male  □ Female

Nationality □ Saudi  □ Non-Saudi

Years of Clinical Experience □ <5  □ 5-10  □ 11-20  □ >20

Practice Type □ Government  □ Private  □ University/Academic

Specialty □ General Dentist  □ Endodontist  □ Others

Section 2: Knowledge and Practice Patterns

Are you familiar with bioceramic sealers? □ Yes  □ No

How would you rate your knowledge of bioceramic sealers? □ Poor  □ Fair  □ Good  □ Excellent

Which of the following do you consider to be advantages of bioceramic sealers? (Select all that apply) ☐ Biocompatibility  ☐ Antimicrobial effect  ☐ Dimensional stability ☐ Ease of use  ☐ Ability to set in moisture

Do you currently use bioceramic sealers in your practice? □ Yes  □ No

If yes, what brand do you use?

How often do you use bioceramic sealers compared to other sealers? □ Always  □ Often  □ Sometimes  □ Rarely

What obturation technique do you most commonly use with bioceramic sealers? □ Single cone  □ Warm vertical compaction  □ Lateral condensation  □ Other: __________

In your opinion, bioceramic sealers provide superior clinical outcomes compared to other sealers. □ Strongly agree  □ Agree  □ Neutral  □ Disagree  □ Strongly disagree

Main barriers to using bioceramic sealers: (Select all that apply) ☐ High cost  ☐ Limited availability  ☐ Lack of training ☐ Unfamiliarity with product  ☐ Satisfactory results with other sealers

Have you attended any continuing education/training programs about bioceramic sealers? □ Yes  □ No
